# Phase I clinical trial to evaluate the safety, tolerability, and pharmacokinetics of high-dose intravenous ascorbic acid in patients with advanced cancer

**DOI:** 10.1007/s00280-013-2179-9

**Published:** 2013-05-14

**Authors:** Christopher M. Stephenson, Robert D. Levin, Thomas Spector, Christopher G. Lis

**Affiliations:** 1 Cancer Treatment Centers of America®, 2520 Elisha Ave., Zion, IL 60099 USA; 2 Cancer Treatment Centers of America®, 1336 Basswood Road, Schaumburg, IL 60173 USA; 3 Spector Consulting Services, 5216 Old Chapel Hill Road, Durham, NC 27707 USA

**Keywords:** Ascorbic acid, Cancer, Pharmacokinetics, Phase I clinical trial

## Abstract

**Purpose:**

This phase I clinical trial evaluated the safety, tolerability, and pharmacokinetics of high-dose intravenous (i.v.) ascorbic acid as a monotherapy in patients with advanced solid tumors refractory to standard therapy.

**Methods:**

Five cohorts of three patients received i.v. ascorbic acid administered at 1 g/min for 4 consecutive days/week for 4 weeks, starting at 30 g/m^2^ in the first cohort. For subsequent cohorts, dose was increased by 20 g/m^2^ until a maximum tolerated dose was found.

**Results:**

Ascorbic acid was eliminated by simple first-order kinetics. Half-life and clearance values were similar for all patients of all cohorts (2.0 ± 0.6 h, 21 ± 5 dL/h m^2^, respectively). *C*_max_ and AUC values increased proportionately with dose between 0 and 70 g/m^2^, but appeared to reach maximal values at 70 g/m^2^ (49 mM and 220 h mM, respectively). Doses of 70, 90, and 110 g/m^2^ maintained levels at or above 10–20 mM for 5–6 h. All doses were well tolerated. No patient demonstrated an objective antitumor response.

**Conclusions:**

Ascorbic acid administered i.v. at 1 g/min for 4 consecutive days/week for 4 weeks produced up to 49 mM ascorbic acid in patient’s blood and was well tolerated. The recommended dose for future studies is 70–80 g/m^2^.

## Background

Preclinical studies of large doses of ascorbic acid (vitamin C) have been reported to show significant anticancer effects in animal models and tissue culture investigations [1–13]. These include direct cytotoxic effects in certain cancer cell lines at micromolar (μM) to millimolar (mM) concentrations [9]. Early clinical studies suggested that intravenous (i.v.) and oral ascorbic acid may diminish symptoms and possibly prolong survival in terminal cancer patients [14–17]. However, more recent double-blind, placebo-controlled studies conducted at the Mayo Clinic have shown that oral administration of ascorbic acid provides no consistent benefit to cancer patients [18, 19].

To date, three phase I clinical trials of i.v. ascorbic acid have been conducted in patients with advanced cancer [20–22]. The first of these trials conducted by Riordan et al. [22] in terminal cancer patients found i.v. ascorbic acid to be relatively safe, provided the patient does not have a history of kidney stone formation. The second trial conducted by Hoffer et al. [20] in advanced cancer patients found that high-dose i.v. ascorbic acid was well tolerated but failed to demonstrate anticancer activity. Finally, the most recently published trial by Monti et al. [21] in metastatic pancreatic cancer patients revealed no increased toxicity with the addition of i.v. ascorbic acid to gemcitabine and erlotinib.

Recent pharmacologic modeling revealed that orally administered ascorbic acid, even at very large and frequent dosing, will increase plasma concentrations only modestly, from 0.07 mM to a maximum of 0.22 mM [23]. Conversely, intravenous ascorbic acid administration can raise plasma concentrations as high as 14 mM, and concentrations of 1–5 mM have been found to be selectively cytotoxic to tumor cells in vitro [1, 2, 9]. Pharmacokinetic studies [24] of i.v. ascorbic acid by Riordan et al. indicated that a 30-g infusion was not adequate to raise plasma levels of ascorbic acid to a level toxic to tumor cells. Infusions of 60 g resulted in brief elevations to 24 mM, while a loading infusion of 60 g followed by an additional 20 g infused over 60 min resulted in a 240-min span in which ascorbic acid plasma concentrations were ≥24 mM. At these time points, serum from patients could be added to in vitro cultures and demonstrates antitumor toxicity [24].

In the hundreds of patients receiving ascorbic acid that have been reported in the literature, only a few have achieved an anticancer response [14]. Given the above observations, one possible explanation is that the concentration of ascorbic acid achieved within the systemic compartment was not adequate. Alternatively, the duration of elevated ascorbic acid concentrations was not sufficient to attain an anticancer response. Therefore, we conducted a phase I clinical trial to determine the safety and tolerability of i.v. ascorbic acid at doses that could potentially achieve the serum concentrations for cytotoxic anticancer activity in patients with solid tumors that might be associated with therapeutic efficacy.

## Methods

### Eligibility criteria

Eligible patients were required to have measurable biopsy-proven advanced and/or metastatic solid tumors that were progressing with no other available known beneficial treatments. Patients were required to be ≥18 years of age, have not received antineoplastic therapy for ≥4 weeks prior to their first dose of i.v. ascorbic acid, have an Eastern Cooperative Oncology Group (ECOG) performance status of 0–2, a life expectancy of ≥3 months, normal glucose-6-phosphate dehydrogenase (G6PD) activity, normal renal function (serum creatinine of <1.2 mg/dl), no evidence of oxalosis by urinalysis, no evidence of chronic hemodialysis, iron overload (serum ferritin >500 ng/ml), Wilson’s disease, or compromised liver function (serum bilirubin >2; AST >63, ALT >95). Pregnant or lactating females, or patients with current tobacco use, evidence of significant psychiatric disorder by history or examination that would prevent completion of the study or preclude informed consent, current aspirin (exceeding 325 mg/day) or acetaminophen (exceeding 2 g/day) use, or the presence of brain metastases that had not responded to therapy were not eligible for the study entry. The protocol was approved by the US Food and Drug Administration under an Investigational New Drug Application. In addition, the protocol was approved by the institutional review board of Cancer Treatment Centers of America^®^ at Midwestern Regional Medical Center, and written informed consent was obtained for each patient prior to performing study-related procedures. An independent Data Safety Monitoring Board was utilized to periodically review and evaluate patient safety, accumulate study data, study progress, and when appropriate make recommendations about continuing or modifying the study.

### Study design

This was a non-comparative, single-center, phase I dose-escalation trial designed to evaluate the safety, tolerability, and pharmacokinetics of high-dose i.v. ascorbic acid in patients with advanced solid tumors who did not respond to standard therapy. A central line was required for the delivery of the i.v. ascorbic acid. Prior to receiving the therapeutic dose, on Day 1, all patients received a test dose of i.v. ascorbic acid of 15 g to ensure that the patient was not hypersensitive to ascorbic acid. After successfully completing the test dose, patients started their trial dose the following Monday. Cohorts of three patients received i.v. ascorbic acid starting at 30 g/m^2^ in the initial cohort. For subsequent cohorts, the dose of ascorbic acid was to be increased by 20 g/m^2^ until a maximum tolerated dose (MTD) or an optimal dose was found. Ascorbic acid was administered at 1 g/min for 4 consecutive days/week for 4 consecutive weeks. If toxicity was encountered at one dose level, the number of patients enrolled would be expanded to 6 or 9 until the toxicity of that dose was determined. If a dose-limiting toxicity (DLT) was observed in ≥2 patients out of 6, the MTD was defined as the dose just below the dose where the DLT was observed. DLT was defined as any reversible Grade ≥3 adverse event, whether hematologic or non-hematologic. If patients treated at the highest-dose cohort (130 g/m^2^) did not experience DLT, investigators had the option of (1) treating another cohort with a higher dose or (2) selecting the ‘treatment dose’ from the dose that provided extended plasma levels of 10–20 mM ascorbic acid. Each cohort received a fixed dose of ascorbic acid for the entire study. Intrapatient dose escalation was not permitted. Concomitant treatment with supportive medications or nutritional interventions was determined by discussion between the principal investigator and the patient’s primary oncologist. For general nutritional support, all patients were provided a multivitamin (Immunomax) along with EPA 2,000 mg daily prior to i.v. ascorbic acid administration.

Ascorbic acid was purchased from Luitpold Pharmaceuticals, Inc/American Regent Laboratories, Inc. Each milliliter contains 0.5 g sodium ascorbate, 0.025 % edetate disodium, and water for injection qs pH (range 5.5–7.0) adjusted with sodium bicarbonate. Ascorbic acid administration conformed to the recommendations provided by Riordan et al. [25]. A clinical pharmacist prepared ascorbic acid within 2 h of its delivery to ensure the sterility of the solution. The solution to be infused contained the appropriate amount of ascorbic acid in sterile water for injection with calcium chloride, magnesium chloride, and potassium chloride. The minerals in solution were in the chloride form because clinical experience with i.v. ascorbic acid has shown a chloride shift, which results in hypochloremia that must be compensated. Ascorbic acid was administered via a central venous access catheter at a rate of 1 g/min because the solution tends to be hyperosmolar (1,200 mOsm/L) and difficult to tolerate in a peripheral vein. The infusion bag of ascorbic acid was protected from light to prevent photooxidation.

### Patient monitoring

Physical examination, height and weight, vital signs and assessments of laboratory analysis (urine analysis, blood chemistry, CBC, screening for G-6-PD and ferritin), and baseline EKG were performed prior to entrance into the study as well as once each week prior to the infusion.

### Pharmacokinetic sampling and assay

Blood samples were taken immediately before the infusion, mid-infusion, and at the infusion endpoint, and 1, 3, 6, and 12 h after the endpoint. Blood samples for PK analyses were obtained on the first and fourth day of the first and fourth week of dosing. Due to early patient withdrawal, fewer data were obtained from patients treated with 110 g/m^2^. Data were not obtained on Week 4, Days 1 and 4 for two patients and on Week 4, Day 4 for one patient in this cohort. Patients doing well could elect to continue being treated 2 days/week on this study protocol after they had completed the initial 4-week program. The initial infusion solution components are presented in Table 1.

**Table 1:** Ascorbic acid was originally prepared according to the following guidelines

Ascorbic acid dose (g)	Sterile water (mL)	Calcium chloride (mEq)	Magnesium chloride (g)	Potassium chloride (mEq)	Final volume
<75	700	54.4	3	20	1,000
75–100	800	68	4	30	1,200
100–125	1,000	85	5	37.5	1,400
125–150	1,200	102	6	45	1,600
150–175	1,400	119	7	52.5	2,000
175–200	1,600	136	8	60	2,200

Stock solution concentrations are as follows: ascorbic acid (0.5 g/ml), calcium chloride (1.36 mEq/ml), magnesium chloride (200 mg/ml), potassium chloride (2 mEq/ml)

Calcium and magnesium in the infusion solution were later reduced to their normal levels in blood (5 and 2 mEq/L, respectively). In addition, potassium was lowered to 16.7 mEq/L, which delivers 10 mEq/h, an infusion rate that is considered safe for all patients

Ascorbic acid was measured in 3 ml of blood sampled at the indicated times by Lab Corp of North Carolina (CLIA #34D0655059; Ascorbate Assay #001479). PK analysis determined the maximal mM drug concentration in plasma (*C*_max_), the h mM area under the drug–concentration curve (AUC) from infusion start to extrapolated infinite time, clearance, and non-compartmental terminal elimination rate.

### Quality of Life assessment

Quality of Life (QoL) was assessed using the European Organization for the Research and Treatment of Cancer Quality of Life Questionnaire (QLQ-C30), which emphasizes a patient’s capacity to fulfill the activities of daily living. The QLQ-C30 is a 30-item cancer-specific questionnaire that incorporates five functioning subscales (physical, role, cognition, emotional, and social), eight symptom scales (fatigue, pain, nausea/vomiting, dyspnea, insomnia, loss of appetite, constipation, and diarrhea), financial well-being scale, and a global scale (based on two items: global health and global QoL). The raw scores are linearly transformed to give standard scores in the range of 0–100 for each of the functioning and symptom scales. Higher scores in the global and functioning scales and lower scores in the symptom scales indicate better QoL. On an average, a difference of 4–10 points reflects a small change, while a difference of 10–20 points represents a medium change in QoL across all QLQ-C30 subscales [26]. This instrument has been extensively tested for reliability and validity [27–29]. The QLQ-C30 was administered at screening, prior to the first test infusion of 15 g of vitamin C and weekly prior to receiving infusions of vitamin C.

## Results

### Patient characteristics

A total of 17 patients were treated in this phase 1 trial. The demographics of study participants are presented in Table 2.

**Table 2:** Patient characteristics (*N* = 17)

Characteristics	No. of patients
Age, years	
Median = 59	
Range = 40–72	
Sex	
Male	6
Female	11
Stage at diagnosis	
I	5
III	8
IV	4
Stage at study entry	
III	1
IV	16
Type of cancer	
Anus	1
Breast	2
Choroid	1
Colon	4
Ear	1
Liver	1
Lung	1
Pancreas	3
Rectum	1
Skin	1
Small bowel	1

### Incipient changes during the study

Several patients in Cohort II, receiving 50 g/m^2^ ascorbic acid, experienced hypercalcemia and transient hypertension. Our original protocol included large amounts of calcium (54.4 mEq/L), magnesium (30 mEq/L = 3 g/L), and potassium (20 mEq/L) in the ascorbic acid infusion solution. The chloride form of these metals was used to compensate for a chloride shift and hypochloremia that may occur with i.v. ascorbic acid. High levels of calcium were added because ascorbic acid, a chelator of calcium, could cause tremors due to hypocalcemia [25]. However, the association constant for ascorbic acid binding of calcium ions, 2.1 M^−1^, is so weak that the concentration of infused ascorbic acid in the blood is too low to chelate significant amounts of calcium.

Large amounts of magnesium had been added to inhibit the formation of oxalate stones, which were believed to be caused by large i.v. doses of ascorbic acid [25]. However, reports have shown that ascorbic acid does not contribute to renal oxalate stone formation [30, 31]. Therefore, the concentrations of calcium and magnesium in the infusion solution were reduced to the same concentrations that appear in blood (5 and 2 mEq/L, respectively). In addition, potassium was lowered to 16.7 mEq/L, which delivers 10 mEq/h, an infusion rate that is considered safe for all patients. Ascorbic acid remained at 100 g/L and was infused at 1 g/min.

### Pharmacokinetics

**Fig. 1:**
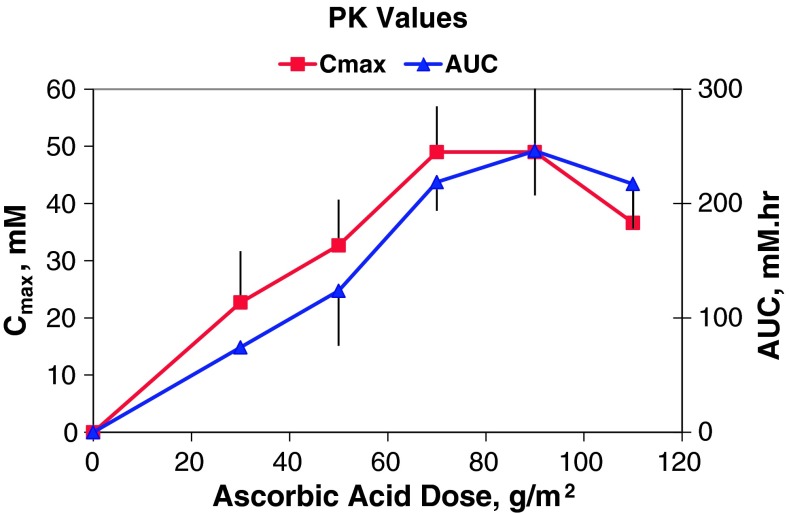
*C*_max_ and AUC values versus ascorbic acid dose

Data were obtained for five cohorts treated with 30, 50, 70, 90, and 110 g/m^2^, respectively. Ascorbic acid was eliminated by simple first-order kinetics. The ascorbic acid elimination half-life (t_**½**_), clearance, *C*_max_, and AUC are presented in Table 3. Ascorbic acid did not accumulate to any significant level during consecutive daily administrations, and the t_**½**_, *C*_max_, and AUC values of ascorbic acid for each patient did not systematically change during their 4 weeks of treatment. The half-life values as well as the clearance values of ascorbic acid were similar for all patients of all cohorts (2.0 ± 0.6 h and 21 ± 5 dL/h m^2^, respectively). The *C*_max_ and AUC values increased proportionately with ascorbic acid dosages between 0 and 70 g/m^2^. The correlation coefficients (*r* ^2^) were 0.99 for the *C*_max_ versus dose and 0.97 for the AUC versus dose for this dosing range (Fig. 1). However, the *C*_max_ and AUC appeared to reach maximum values at 70 g/m^2^.

**Table 3:** Pharmacokinetic values

PK parameters	Ascorbic acid dose (g/m^2^)				
	30	50	70	90	110^a^
Elimination t_**½**_ (hours)	**2.1** ± 0.9^b^ (1.6–3.1)	**1.8** ± 0.4 (1.4–2.2)	**1.7** ± 0.2 (1.5–1.9)	**2.1** ± 0.3 (1.8–2.3)	**2.5** ± 0.9 (1.6–3.3)
Clearance (dL/h m^2^)	**23** ± 0.8 (22–24)	**25** ± 10 (16–36)	**18** ± 2 (16–20)	**18** ± 4 (14–21)	**20** ± 4 (16–23)
*C*_max_ (mM)	**23** ± 9 (8–38)	**33** ± 8 (15–47)	**49** ± 8 (35–60)	**49** ± 14 (37–78)	**37** ± 6 (28–46)
AUC (h mM)	**74** ± 3 (72–77)	**124** ± 50 (80–175)	**219** ± 25 (200–247)	**246** ± 40 (206–285)	**217** ± 40 (174–249)

^a^Incomplete data were obtained for this cohort. Week-4 data were not available for two subjects, and Week-4, Day-4 data were not available for one subject

^b^Average value ± SD (range)

There were no statistical differences (2-tailed Student’s *t* test) between the AUC values of 219, 246, and 217 h mM obtained from the 70, 90, and 110 g/m^2^ ascorbic acid doses, respectively. The 70 and 90 g/m^2^ doses of ascorbic acid achieved a *C*_max_ value of 49 mM, while the *C*_max_ value of the 110 g/m^2^ dose was 37 mM. Although this value was significantly lower (2-tailed Student’s *t* test) than the 49 mM values obtained from the 70 and 90 g/m^2^ dose, fewer data points were collected from this cohort because of early subject discontinuation. The *p* values for comparing the *C*_max_ values were 0.002 (110 vs. 70 g/m^2^) and 0.04 (110 vs. 90 g/m^2^), respectively. Each of the three highest doses (70, 90, and 110 g/m^2^) maintained ascorbic acid blood levels at or above 10–20 mM for about 5–6 h.

### Adverse events

Table 4 describes Grade 2 and above adverse events experienced by patients during the trial stratified by dose cohort. In general, high-dose i.v. ascorbic acid was well tolerated, and most adverse events were mild and only possibly or probably related to the treatment. Treatment-related nausea and headache were fairly common in all cohorts. Some patients had moderate to severe hypernatremia and hypokalemia. Other reported adverse events were hypertension, insomnia, abnormal urine color, loss of appetite, fatigue, chills, and hyperglycemia.

**Table 4:** Number of patients experiencing adverse events

Toxicity	Grade 2	Grade 3	Grade 4
Dose cohort I (*n* = 3)			
Proteinuria	1	0	0
Granular casts	1	0	0
Dose cohort II (*n* = 5)			
Hypertension	2	0	0
Pain—lower back	1	0	0
Tumor fever	1	0	0
Pedal enema	1	0	0
Bacteremia	1	0	0
Hypoalbuminemia	1	0	0
Hypokalemia	1	0	0
Peripheral neuropathy	1	0	0
Dose cohort III (*n* = 3)			
No adverse effects			
Dose cohort IV (*n* = 3)			
Hypokalemia	0	2	0
Hypernatremia	0	0	2
Hypertension	1	0	0
Headache	0	1	0
Hyperglycemia	1	0	0
Dose cohort V (*n* = 3)			
Headache	1	0	0
Hypertension	1	0	0
Hypernatremia	0	1	1
Increased LDH	1	0	0
Anemia	2	0	0
Hypercalcemia	1	0	0
Increased creatinine	1	0	0

Cohorts I–V represent 30, 50, 70, 90, and 110 g/m^2^ doses of ascorbic acid, respectively

### Antitumor efficacy

One patient was withdrawn from the study and hence could not be evaluated for tumor response. Of the remaining 16 patients, no one experienced an objective tumor response. Three patients had stable disease, while 13 had progressive disease.

### Quality of life

Table 5 displays the QLQ-C30 scores over the entire course of the study. The average values for the functioning and symptom scales remained fairly constant for 2 weeks and then appeared to improve for the fewer patients who completed the questionnaire at 3 and 4 weeks.

**Table 5:** Quality of life assessment

Visit	Screening	Prior to test infusion	Week 1	Week 2	Week 3	Week 4
Sample size	*N* = 17	*N* = 17	*N* = 16	*N* = 12	*N* = 7	*N* = 2
Functioning scales (higher = better)						
Global	69	58	59	65	74	92
Physical	79	69	73	68	72	87
Role	68	52	65	61	67	100
Emotional	70	77	76	88	89	100
Cognitive	75	75	81	85	86	83
Social	60	64	55	69	71	75
Symptoms (lower = better)						
Fatigue	41	49	46	40	30	11
Nausea/vomiting	19	27	18	32	14	0
Pain	39	36	35	35	29	0
Dyspnea	20	24	25	19	29	0
Insomnia	25	31	35	28	29	17
Appetite loss	39	41	44	33	10	17
Constipation	25	31	33	31	14	33
Diarrhea	14	24	10	3	0	0
Financial problems	45	33	33	17	38	50

## Discussion

A previous phase I ascorbic acid trial [20] stopped dose escalation at 1.5 g/kg (approximately 56 g/m^2^) when peak blood levels approached 26 mM, the level that inhibited tumor growth in mice [3]. However, 30–40 mM ascorbic acid in mouse blood only partially inhibited the rate of tumor growth and did not produce tumor regression. Our study achieved higher blood levels, longer drug exposures, and higher dose intensity to better assess both toxicity and the potential for antitumor efficacy. These parameters are compared for the two studies in Table 6. Our protocol produced approximately a twofold higher *C*_max_ and AUC values for ascorbic acid in patient’s blood, reaching values of 49 mM and 246 h mM, respectively. Dose levels of 70, 90, and 110 g/m^2^ also maintained ascorbic acid blood levels at or above 10–20 mM for about 5–6 h. These high concentrations of ascorbic acid (both peak and sustained) were generally well tolerated.

**Table 6:** Phase I studies compared

Variable	Hoffer et al. [20]	CTCA
Schedule	3 days per week	4 days per week
Highest dose	56 g/m^2^ (1.5 g/kg)^a^	110 g/m^2^
Highest dose intensity	168 g/m^2^/week	440 g/m^2^/week
Highest *C*_max_	26 mM	49 mM
Highest AUC	Approx. 100 mM h	246 mM h

^a^g/kg × 37 = g/m^2^ [37]

Although the *C*_max_ and AUC values for ascorbic acid increased proportionately with ascorbic acid doses between 0 and 70 g/m^2^, the values of these parameters did not increase much further at the higher doses of 90 and 110 g/m^2^. Therefore, a dose of 70–80 g/m^2^ appears to be optimal for future studies since higher doses provide no additional drug-exposure benefits and subject the patient to unnecessarily longer infusions.

The 2008 report of Heaney et al. [32] advised caution concerning combining ascorbic acid with other chemotherapeutic agents. Using hematopoietic cell cultures and hematopoietic xenogenic tumors in mice, they studied the effects of ‘vitamin C’ on the antitumor activity of several common chemotherapeutic agents (doxorubicin, cisplatin, vincristine, methotrexate, and imatinib), including some agents that do not have reactive oxygen mechanisms of action. They found that ascorbate at higher concentrations antagonized the cytotoxicity of some chemotherapeutic agents, and they concluded that ‘vitamin C’ might interfere with the treatment for hematopoietic tumors (and other tumor types) in cancer patients. Unfortunately, they used dehydroascorbic acid (not ascorbic acid) and claimed that it was converted to ascorbic acid intracellularly. A particular concern with this study is that one of the more likely potential antitumor mechanisms of action of ascorbic acid depends on the extracellular conversion of ascorbic acid to dehydroascorbic acid, which generates extracellular H_2_O_2_, an active cytotoxic antitumor agent [3, 33]. Thus, dosing with dehydroascorbic acid may circumvent this particular antitumor activity of ascorbic acid. This probability is supported by their findings that 8.5–18 mM dehydroascorbic acid alone produced no antitumor activity against their two cell lines and that 250 mg/kg dehydroascorbic acid produced only minimal antitumor activity against the xenographic tumors in mice. Similar criticism of the conclusions of this report has been voiced by others [34].

Further support for a potential role of i.v. ascorbic acid is provided by the work of Verrax and Calderon [33] who demonstrated that ascorbic acid completely kills a variety of tumor cells, T24 (bladder), DU145 (prostate), HepG2 (liver), MCF7 (breast), and Ishikawa (cervix), with EC_50_ values of 3–7 mM. They also showed that 1 g/kg/d i.p. ascorbic acid significantly inhibited TLT tumor growth in mice without any obvious toxicity. Notably, orally dosed ascorbic acid had no effect on tumor growth. In addition, this group demonstrated that ascorbic acid significantly potentiated the antitumor activity of several chemotherapeutic agents including etoposide, cisplatin, 5-fluorouracil, doxorubicin, and paclitaxel and in all three tumor lines tested (MCF7, DU145, and T24).

The use of i.v. ascorbic acid in combination with cytotoxic chemotherapy is further encouraged by a recent report showing that ascorbic acid potentiated the antitumor activity of gemcitabine against seven human and one murine pancreatic cancer cell lines [35]. The observation is of particular interest as two of the human lines were resistant to gemcitabine. Synergistic antitumor activity occurred in tissue culture and in vivo studies with implanted tumors in mice. This and other reported preclinical investigations provide encouragement for additional exploration of i.v. ascorbic acid to improve therapeutic outcomes [36]. A recently published phase I clinical trial by Monti et al. evaluated i.v. ascorbate combined with gemcitabine and erlotinib in nine patients with stage IV metastatic pancreatic cancer. Potentially biologically and clinically active ascorbic acid concentrations were achievable in all treated individuals, and primary tumor size decreased in eight out of nine patients [21]. The findings of the current study coupled with the collective evidence from the available literature suggest that the combination of i.v. ascorbic acid with gemcitabine to treat pancreatic cancer is an attractive approach that deserves further evaluation.

## Conclusions

In conclusion, ascorbic acid dosed i.v. at 1 g/min for 4 consecutive days/week for 4 weeks resulted in a peak concentration of approximately 49 mM and was generally well tolerated. The recommended dose for future studies is 70–80 g/m^2^.
